# Metabolism, Disposition, Excretion, and Potential Transporter Inhibition of 7–16, an Improving 5-HT_2A_ Receptor Antagonist and Inverse Agonist for Parkinson’s Disease

**DOI:** 10.3390/molecules29102184

**Published:** 2024-05-08

**Authors:** Zhengping Hu, Wenyan Wang, Huijie Yang, Fengjuan Zhao, Chunjie Sha, Wei Mi, Shuying Yin, Hongbo Wang, Jingwei Tian, Liang Ye

**Affiliations:** 1 Medicine and Pharmacy Research Center, Binzhou Medical University, Yantai 264003, China; 2 School of Pharmacy, Key Laboratory of Molecular Pharmacology and Drug Evaluation (Yantai University), Ministry of Education, Collaborative Innovation Center of Advanced Drug Delivery System and Biotech Drugs in Universities of Shandong, Yantai University, Yantai 264005, China; wangwenyan@luye.com (W.W.);; 3 State Key Laboratory of Advanced Drug Delivery and Release Systems, Shandong Luye Pharmaceutical Co., Ltd., Yantai 264003, Chinazhaofengjuan@luye.com (F.Z.);; 4 School of Public Health, Binzhou Medical University, Yantai 264003, China

**Keywords:** metabolite identification, metabolic enzyme phenotype, excretion, UPLC-Q Exactive HRMS, radioanalysis, Parkinson’s disease psychosis

## Abstract

Compound 7–16 was designed and synthesized in our previous study and was identified as a more potential selective 5-HT_2A_ receptor antagonist and inverse agonist for treating Parkinson’s disease psychosis (PDP). Then, the metabolism, disposition, and excretion properties of 7–16 and its potential inhibition on transporters were investigated in this study to highlight advancements in the understanding of its therapeutic mechanisms. The results indicate that a total of 10 metabolites of 7–16/[^14^C]7–16 were identified and determined in five species of liver microsomes and in rats using UPLC-Q Exactive high-resolution mass spectrometry combined with radioanalysis. Metabolites formed in human liver microsomes could be covered by animal species. 7–16 is mainly metabolized through mono-oxidation (M470-2) and N-demethylation (M440), and the CYP3A4 isozyme was responsible for both metabolic reactions. Based on the excretion data in bile and urine, the absorption rate of 7–16 was at least 74.7%. 7–16 had weak inhibition on P-glycoprotein and no effect on the transport activity of OATP1B1, OATP1B3, OAT1, OAT3, and OCT2 transporters. The comprehensive pharmacokinetic properties indicate that 7–16 deserves further development as a new treatment drug for PDP.

## 1. Introduction

Parkinson’s disease psychosis (PDP) includes nonmotor symptoms experienced by almost 60% of individuals suffering from Parkinson’s disease, such as autonomic dysfunction, sensory disturbances, and neuropsychiatric manifestations including hallucinations, delusions, cognitive impairment, sleep disturbances, depression, and anxiety [1,2]. No antipsychotic drug had been indicated for PDP until the emergence of pimavanserin, a selective 5-HT_2A_ receptor antagonist and inverse agonist, which was approved for listing in the USA in 2016 [3]. However, it’s reported that pimavanserin was associated with a potential risk of prolonged QT interval in patients [4,5]. In our previous study [6], a series of novel pimavanserin derivatives were designed and synthesized to improve its 5-HT_2A_ receptor antagonist and inverse agonist activity so as to reduce clinical dosage. Fortunately, we found that compound 7–16 exhibited 50-fold higher 5-HT_2A_ receptor antagonist activity (IC_50_ = 0.54 nM) and 23-fold higher inverse agonist activity (IC_50_ = 2.10 nM) than pimavanserin (IC_50_ = 27.3 and 50.0 nM, respectively), as well as decreased hERG inhibitory activity (IC_50_ = 1.10 nM), which was 2-fold weaker than that of pimavanserin. In vivo, it suggested that 7–16 was at least four times more potent than pimavanserin for blocking 5-HT_2A_ receptors by the head twitches test in rats.

The development of safe and effective medications requires a profound understanding of their pharmacokinetic (PK) characteristics, which could be elucidated through investigation of drug absorption, disposition, metabolism, and excretion. Among these, metabolite identification contributes to understanding its pharmacological effects, selecting animal species in toxicology evaluation, and predicting toxicity [7]. Moreover, the in vivo study of metabolism and disposal is more critical in understanding metabolic pathways and rates and routes of excretion, which helps to identify and quantitate the exposure to metabolites and also to predict the safety of the parent drugs [8]. Wu et al. [9] studied and identified the metabolites of pimavanserin in rats, and a total of 23 metabolites were detected and identified using ultra-high performance liquid chromatography (UPLC) combined with Fourier transform ion cyclotron resonance high-resolution mass spectrometry (HRMS). However, the mass spectroscopy signal intensities generally cannot accurately estimate the actual abundance of metabolites in metabolism and excretion relative to the unchanged drug. Radioanalysis combined with UPLC-HRMS can provide accurate quantitative data for identified metabolites, which can lead to a better insight into the pharmacokinetic processes [10,11,12]. In addition, drug–drug interactions (DDIs) mediated by cytochrome P450 enzymes or those efflux/uptake transporters can impact the efficacy or toxicity of medications [13,14].

In this study, we carried out a series of experiments in vivo and in vitro to understand the PK characteristics of 7–16, including metabolite identification of 7–16 in liver microsomes of different species using UPLC-Q Exactive HRMS and a metabolism and excretion study of 7–16 in rats after oral administration of carbon 14 (^14^C) labeled 7–16 ([^14^C]7–16) by UPLC-Q Exactive HRMS and LC coupled with radioactivity monitor (LC-RAM). Meanwhile, the metabolic enzyme phenotype of 7–16 in human recombinant enzymes and the inhibition potential of 7–16 on the transport activity of several main efflux/uptake transporters are closely related to clinical DDIs, such as P-glycoprotein (P-gp), organic anion transporting polypeptides 1b1 and 1b3 (OATP1B1 and OATP1B3), organic anion transporters 1 and 3 (OAT1, OAT3), and organic cation transporter 2 (OCT2). These study results provided data support for the further development of 7–16.

## 2. Results and Discussion

### 2.1. Metabolite Identification in Liver Microsomes

**Figure 1:**
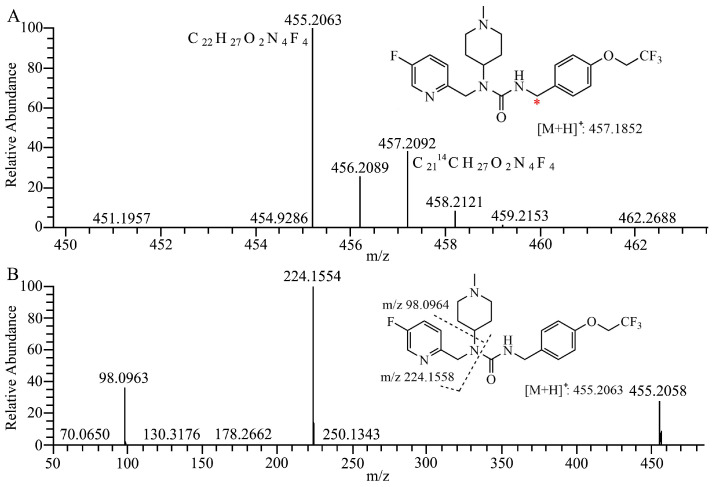

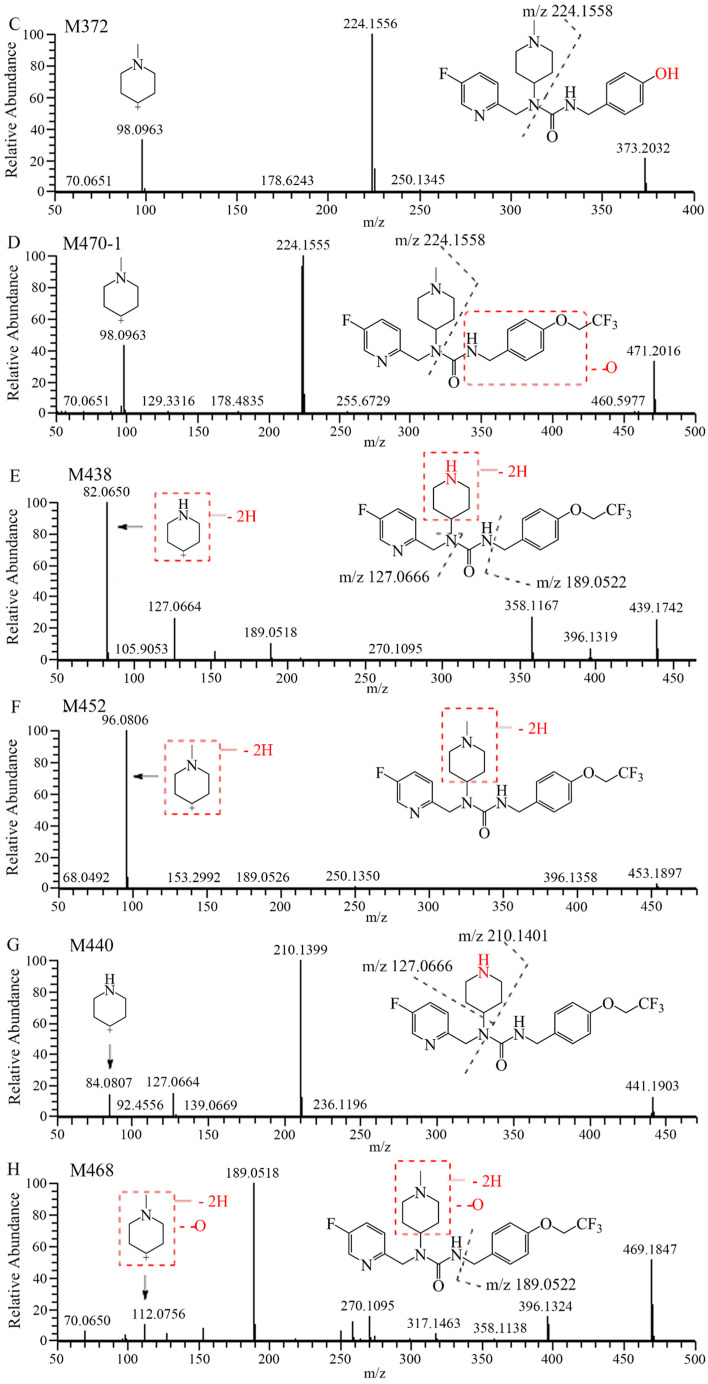

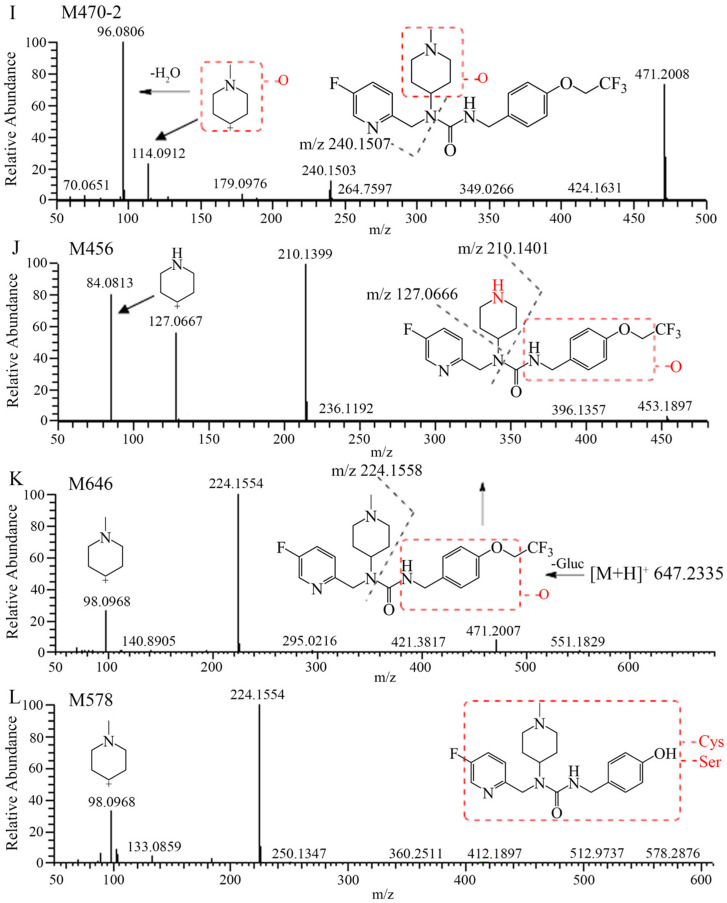
Structures and full scan mass spectrum of 7–16 and [^14^C]7–16 (**A**) * ^14^C labeling site, product–ion spectra and proposed fragmentation pathways of 7–16 (**B**) and its metabolites (**C**–**L**).

As presented in Figure 1A, the [M + H]^+^ ion of 7–16 and its ^14^C labeled compound [^14^C]7–16 is observed at *m*/*z* 455.2054 (−2.4 ppm) and *m*/*z* 457.2097 (−1.1 ppm), respectively. The fragment ion of 7–16 is presented in Figure 1B, and fragment ions at *m*/*z* 224 and *m*/*z* 98 (N-methylpiperidine) are the main references for the analysis of metabolic sites of metabolites.

The mass spectra, retention times, and relative abundance of 7–16 and its detected metabolites after incubation in liver microsomes from humans, mice, rats, dogs, and monkeys have been summarized in Table 1. The combined fragment ion mass analysis of 7–16 and its metabolites, as presented in Figure 1B–I, are identified as seven metabolites, including the O-dealkylation metabolite (M372), mono-oxidation metabolites (M470-1, M470-2), demethylation and dehydrogenation metabolite (M438), dehydrogenation metabolite (M452), N-demethylation metabolite (M440), and mono-oxidation and dehydrogenation metabolite (M468). M372 is identified as the loss of the trifluoroethyl group, decreasing its mass to 82 (C_2_HF_3_) compared with 7–16. M470-2, with the fragment ions of *m*/*z* 240 and *m*/*z* 114, features the increased oxygen molecules (16) from the fragment ions of 7–16 at *m*/*z* 224 and *m*/*z* 98, indicating that the oxidation occurred at the N-methylpiperidine group. M470-1 is oxidized at the trifluoroethoxy-benzy group because there were the same fragment ions of *m*/*z* 224 and *m*/*z* 98 as that of 7–16. M440 is identified as the demethylation metabolite, decreasing a mass loss of 14 compared with 7–16, with the characteristic fragment ions at *m*/*z* 210, *m*/*z* 127, and *m*/*z* 84. Furthermore, M438 and M452 produced characteristic fragment ions of *m*/*z* 82 and *m*/*z* 96, respectively, indicating that the dehydrogenation occurred in the piperidine group. Based on the presence of the characteristic fragment ions of *m*/*z* 189 and *m*/*z* 112, the dehydrogenation and oxidation site of the M468 is ascertained at the N-methylpiperidine group.

After incubation of liver microsomes of five species, the parent drug accounted for the main proportion, with a relative abundance of 77.2–95.2%. M470-2 was the main metabolic product with a relative abundance of 2.53–17.3%. The relative abundances of other metabolites are observed to be less than 1.92%.

All the metabolites detected in humans are also found in animal species, demonstrating that no unique metabolites are formed in human liver microsomes after incubation at 37 °C for 60 min. The major metabolite M470-2 exhibits a relatively high abundance in animal species as compared with humans, whereas all other metabolites are present in similar proportions in both humans as well as animals, which provides data support for animal species selection for toxicology and safety evaluations of 7–16.

### 2.2. Metabolite Identification in Rat Plasma, Urine, Feces, and Bile

**Figure 2:**
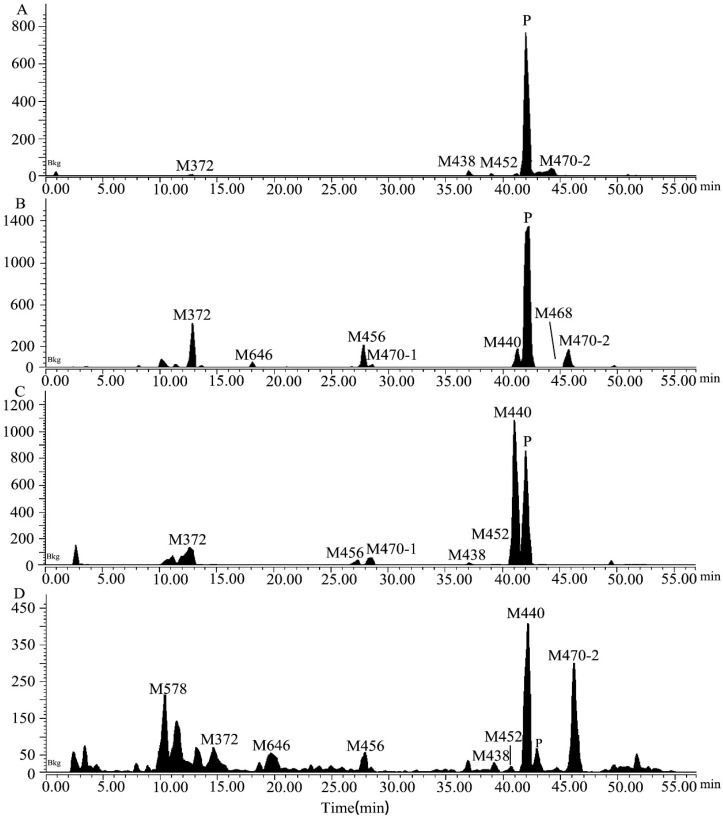
The extracted ion chromatograms in rat plasma (**A**), urine (**B**), feces (**C**), and bile (**D**) samples obtained after a single oral dose of [^14^C]7–16 at 3 mg/100 µCi/kg.

The extraction ion chromatograms and retention times of 7–16 and its metabolites in rat plasma, urine, feces, and bile samples after oral administration have been depicted in Figure 2. Table 2 lists the mass spectrum findings of the [^14^C]7–16 and its metabolites identified in rats. After analysis of the fragment ion mass of 7–16 and its metabolites, besides the seven metabolites found in microsomes (Figure 1C–I), three metabolites were found including the N-demethylation and mono-oxidation metabolite (M456), mono-oxidation and glucuronide conjugate (M646), and dealkylation and conjugated cysteine and serine (M578) (Figure 1J–L). The latter two are mainly found in bile. M456 has the same fragment ions (*m*/*z* 210, *m*/*z* 127, and *m*/*z* 84) as M440, so it was oxidized at the trifluoroethoxy-benzy group. M646 increased its mass 176 more than M470, and it had the fragment ions of *m*/*z* 224 and *m*/*z* 98, so it was oxidized and conjugated glucuronide at the trifluoroethoxy-benzy group. M578 had a mass increase of 206 compared with M372, having the same fragment ions (*m*/*z* 224 and *m*/*z* 98) as M372. Through accurate quality calculations, it was speculated by Thermo Compound Discover software (Version 3.3) that M578 was produced by M372 conjugated cysteine and serine.

The relative abundances of the metabolites were calculated from the percentage of total radiation intensity (TRA%) in plasma and from the percentage of dose in urine, feces, and bile. The results indicate the sex-based differences in exposure to 7–16 in rats, shown in Table 2. The relative abundance of unchanged [^14^C]7–16 in female and male rat plasma is 81.9% and 57.3%, respectively, and accounts for most of the content. Meanwhile, M470-2 is the main metabolite detected in the plasma, with relative abundances of 8.63% and 27.2% in females and males, respectively. In urine, [^14^C]7–16 is also dominant, with abundances of 23.6% and 9.79% observed in females and males, respectively, followed by metabolite M440, which is the predominant metabolite in feces (21.1% in females and 24.2% in males). Hence, M470-2 and M440 are major metabolites detected in vivo.

**Figure 3:**
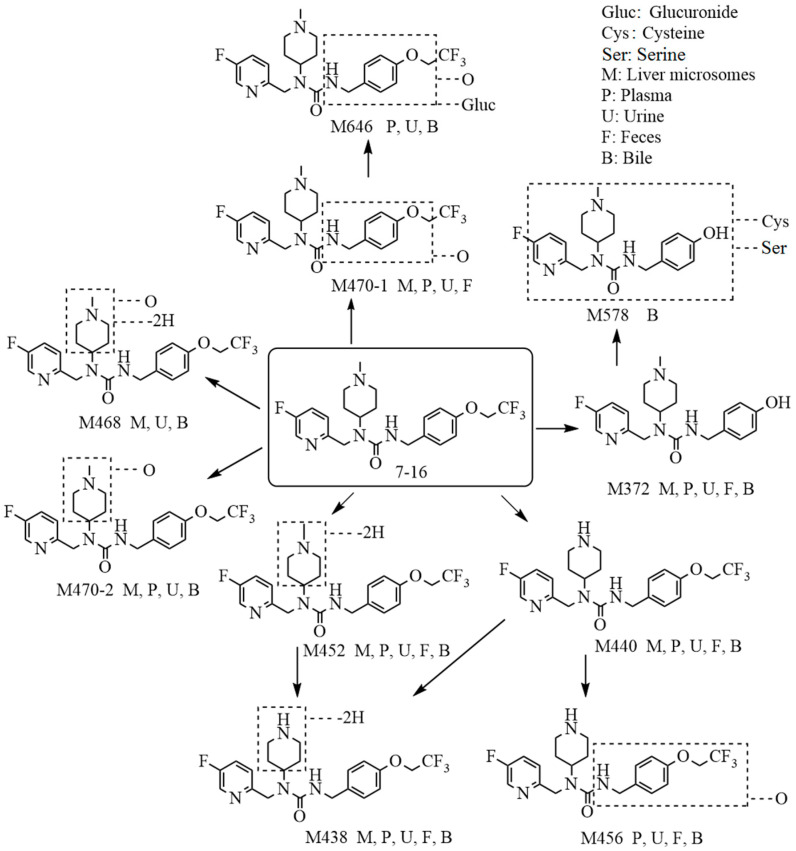
The proposed metabolic pathways of 7–16 in liver microsomes and in rats.

A total of 10 metabolites of 7–16 are identified in vitro and in vivo, and 7–16 is considered to undergo metabolism through two pathways, namely, mono-oxidation and N-demethylation. The analysis revealed that 7–16 is metabolized by phase I reactions in vivo, and the metabolites formed are excreted through urine and feces. The proposed metabolic scheme is depicted in Figure 3. Compared with pimavanserin, the introduction of the trifluoroethyl group not only significantly improved the activity of the compound but also greatly enhanced its stability [6,15].

### 2.3. Metabolic Phenotype by P450 Enzymes

**Figure 4:**
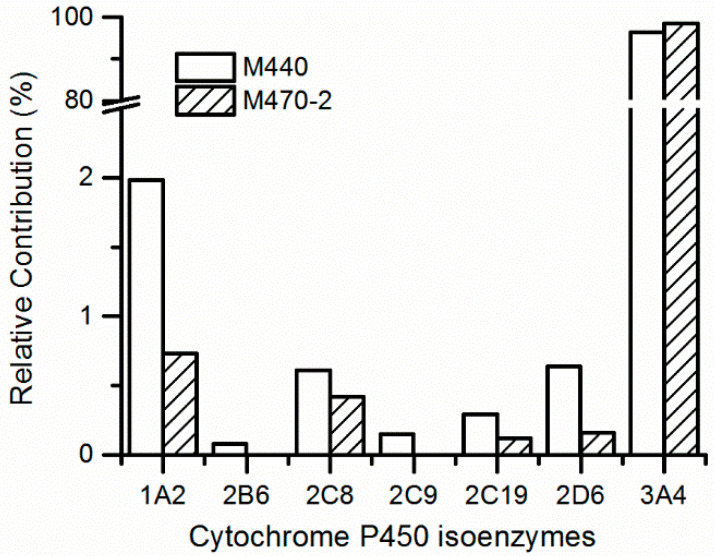
Prediction of relative contribution of cytochrome P450 isoenzymes to 7–16 metabolites in human liver microsomes.

In accordance with the metabolite identification results, M470-2 and M440 were selected as the targets for metabolic phenotype study using recombinantly expressed human cytochrome P450 (rhCYP) isozymes. The results indicate that CYP3A4 is the primary isozyme responsible for the mono-oxidation and N-demethylation metabolism of 7–16, as evident from Figure 4. The relative contributions of CYP3A4 range from 93.2% to 96.7%. Meanwhile, there is little or no contribution from CYP1A2, CYP2B6, CYP2C8, CYP2C9, CYP2C19, and CYP2D6 isozymes. The clearance data of the probe substrates of seven rhCYP isozymes are all normal and acceptable.

Phenotypic studies of metabolic responses contribute to comprehending the roles of the P450 enzyme in drug metabolism processes [16,17]. Human hepatic CYP3A4, recognized as a multifunctional enzyme, has a wide range of substrates, including commonly used drugs [18]. 7–16 is a substrate of CYP3A4, and the DDIs might be provoked by co-administration of drugs that are potent inhibitors or inducers of the enzyme, which should be paid more attention to in future clinical trials of 7–16. In our previous study [6], 7–16 was demonstrated as a medium inhibitor of CYP1A2 with an IC_50_ value of 8.5 μM and almost no inhibition for CYP2C9, CYP2C19, CYP2D6, and CYP3A4 with an IC_50_ value more than 62.9 μM. Although it will depend on the dose applied, the potential cause of DDIs due to inhibition of P450 enzymes by 7–16 is very low.

### 2.4. Excretion in Rats

**Figure 5:**
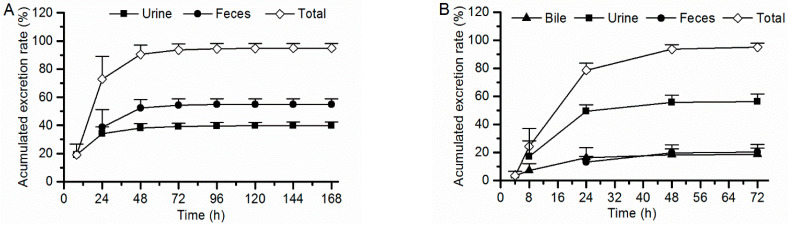
Accumulated excretion rate of radioactivity in rat excreta following a single oral dose of [^14^C]7–16 at 3 mg/100 µCi/kg to intact rats ((**A**), *n* = 6) and bile duct cannulated rats ((**B**), *n* = 8).

Female and male rats for both intact rats and BDC rats exhibit similar excretion ratios of 7–16 after a single oral dose (the percent difference is less than 6.5% between the two sexes), and the mean values of the recovery of radioactivity in excreta (urine, feces, and bile) from both sexes were used as described below, which has been expressed as a percent of the administered dose, shown in Figure 5. For intact rats, a total of 98.9% of the administered drug was recovered within 0–168 h post-administration, with 39.9% of the drug recovered from the urine, 55.0% in feces, and 4.06% in cage rinse/wash. Most of [^14^C]7–16 (76.8% of the dose) were recovered within the first 24 h post-administration. For BDC rats, 98.3% of the dose was recovered within 0–72 h post-administration, with 56.3% recovered in urine, 20.2% in feces, 18.4% in bile, and 3.42% in cage rinse/wash. Most of the radioactivity (82.8% of the dose) was recovered within the first 24 h post-administration. Based on the percentage of the dose excreted in bile and urine from BDC rats, the absorption rate of 7–16 was calculated to be at least 74.7% of the dose. Thus, 7–16 can be considered to display excellent absorption properties.

### 2.5. Inhibitor Assay for the P-gp, OATP1B1, OATP1B3, OAT1, OAT3, and OCT2 Transporters

The results show that 7–16 inhibits P-gp mediated efflux of digoxin with an IC_50_ value of 30.6 µM, as shown in Table 3. At the tested conditions, the percent of lucifer yellow in every basolateral well is no more than 0.4 (acceptance standards are less than 2.0), which indicates a good MDR1-MDCK II monolayer integrity. And verapamil inhibits P-gp activity with an IC_50_ of 1.57 µM, indicating that the test system is considered acceptable. In addition, it was demonstrated that 7–16 does not inhibit the transport activity of OATP1B1, OATP1B3, OAT1, OAT3, and OCT2 with IC_50_ values all more than 72.5 µM. For positive inhibitors, the calculated IC_50_ values are acceptable (Table 3), which indicates that the test system is inhibitable.

P-gp is one of the most important and well-studied efflux transporters. It is expressed in many tissues, including the intestine, liver, kidney, and brain, with the functions of actively pumping small molecule compounds out of cells [19]. On another side, influx transporters have the important role in small-molecule-compound transport across hepatocytes (OATP1B1, OATP1B3) and kidney cells (OAT1, OAT3, and OCT2) [20,21]. In addition, transporter inhibition plays a role that cannot be underestimated in a number of side effects, including an imbalance of endobiotics and dietary nutrients [13,22]. It is very important to understand the impact of 7–16 on the physiologic function of transporters and to take into account these effects in drug discovery and clinical practice. Based on the results of excretion in rats, 7–16 is eliminated via the urine, with a much lesser extent in bile and feces. Thus, the potential to cause DDIs due to transporter inhibition by 7–16 was low. This study provides a database aiding further research and the development of 7–16.

## 3. Materials and Methods

### 3.1. Materials

#### 3.1.1. Chemicals and Reagents

7–16 [3-(4-(2,2,2-trifluoroethoxy)benzyl)-1-((5-fluoropyridin-2-yl)methyl)-1-(1-methylpiperidin-4-yl) urea] (purity > 99%) was offered by the Medicinal Chemistry Research Department, R&D Center (Luye Pharma Group Ltd., Yantai, China). [^14^C]7–16 (chemical purity of 99.50%, and radiochemical purity of 99.57%) was prepared by WuXi AppTec (Shanghai, China) Co., Ltd. Human, rat, mouse, and dog liver microsomes and recombinantly expressed human cytochrome P450 (rhCYP) isozymes were purchased from Corning Co., Ltd. (Shanghai, China) Monkey liver microsomes were purchased from RILD Liver Disease Research Co., Ltd. (Shanghai, China) Substrates of P450 isozymes and their specific metabolites and NADPH were purchased from Sigma-Aldrich (Shanghai, China). HPLC-grade acetonitrile and methanol were purchased from Merck (Darmstadl, Germany). All chemicals and reagents were of analytical grade and purchased from Sinopharm Chemical Reagent (Shanghai, China).

#### 3.1.2. Animals

The adult Sprague-Dawley rats (weighing 200 ± 20 g) were obtained from Weitong Lihua Experimental Animal Technology Co., Ltd. (Jiaxing, China) and were housed in a controlled environment, including temperature (20~26 °C), relative humidity of 40~70%, and a 12 h light/dark cycle. All animals had free access to feed and water in strict accordance with the National Institute of Health Guide for the Care and Use of Laboratory Animals (NIH Publications No. 8023). And the experiment protocols were approved by the Institutional Animal Care and Use Committee.

### 3.2. Methods

#### 3.2.1. Metabolite Identification in Liver Microsomes of Different Species

In accordance with our previous methods [23], 7–16 (10 µM) was incubated with mouse, rat, dog, monkey, and human liver microsome proteins (1 mg/mL) in 100 mM sodium phosphate buffer (pH 7.4) containing 10 mM magnesium chloride and 2 mM NADPH at 37 °C for 60 min. All samples were prepared in duplicates. The reactions were terminated by adding three times the volume of ice-cold acetonitrile (600 µL) and stored at −20 °C until analysis. Control samples without NADPH and 0-time point samples were included. As a positive control, testosterone (10 µM), which was metabolized to produce the hydroxylation metabolite, was used as a substrate to confirm the activity of the incubation system.

After pre-treatments, the samples were analyzed by an Acquity UPLC system coupled with a diode array detector (Waters Corporation, Milford, MA, USA) and Q Exactive HRMS (Thermo Electron Corporation, Waltham, MA, USA) (UPLC–UV-Q Exactive HRMS). Samples were separated on an Agilent XDB C18 column (150 × 2.1 mm i.d. 3.5 μm, Agilent Corporation, Santa Clara, CA, USA) at 40 °C by gradient elution with water and acetonitrile containing 0.1% formic acid. The flow rate was 0.40 mL/min. The ultraviolet (UV) detection wavelength was 286 nm. Q Exactive HRMS were operated at positive ion mode (3.5 kV) using a heating electrospray ionization source. Mass calibration was required to be performed before conducting experiments so as to achieve the best mass accuracy and mass calibration should be performed every five days. The metabolites were detected by full MS/dd-MS^2^ analysis from *m*/*z* 100 to *m*/*z* 1000. The resolution of the first-class scan and second-class scan was 70,000 and 17,500, respectively. Thermo Compound Discover software was used to identify expected and unknown metabolites, and we finally proposed the 7–16 metabolic pathways based on the deduced metabolites and relevant drug biotransformation knowledge.

#### 3.2.2. Metabolite Identification in Rat Plasma

The rats received a single oral dose of [^14^C]7–16 at 3 mg/100 µCi/kg (3 females and 3 males for each time point). At 0.25, 1, 6, and 24 h after administration, about 0.3 mL of blood was collected into a collection vessel containing K_2_-EDTA anticoagulant by cardiac puncture, and the plasma samples were acquired after centrifugation at 8000 rpm for 10 min at 4 °C. All samples were stored at −20 °C until analysis. The rat plasma samples at each time point were mixed in accordance with the area under the concentration curve contribution at corresponding time intervals [6,24]. After the pooled plasma samples were pre-treated, the radio-profiles of plasma samples were detected using Shimadzu LC coupled with a radioactivity monitor (LC-RAM, Scintillation and Luminescence Counter Technology, Perkin Elmer Corporation, Waltham, MA, USA), and the metabolites were identified by UPLC-QE HRMS (Thermo Electron Corporation, Waltham, MA, USA). The percentage of each metabolite based on the total radiation intensity (TRA%) in plasma samples was calculated. The structures of metabolites were identified by the same method used for metabolite identification in the liver microsomes of different species.

#### 3.2.3. Excretion in Rats and Metabolite Identification in Rat Urine, Feces, and Bile

In the urine and feces excretion experiment, six rats (3 females and 3 males) were administered a single oral dose of [^14^C]7–16 at 3 mg/100 µCi/kg. Urine, feces and cage washing/cleaning solution samples were collected from 0 to 168 h following drug administration at the following time intervals: 0–8, 8–24 h (0–24 h for feces), 24–48, 48–72, 72–96, 96–120, 120–144, and 144–168 h. In the biliary excretion experiment, 8 rats using a bile duct cannula (BDC) (4 females and 4 males) were administered a single oral dose of [^14^C]7–16 at 3 mg/100 µCi/kg. Bile, urine, feces, and cage washing solution were collected from before and 0–72 h after oral administration at the following time intervals: 0–4, 4–8 (urine 0–8), 8–24 (feces 0–24), 24–48, and 48–72 h. All samples were collected and refrigerated at −20 °C until determination of the radioactive concentrations by liquid scintillation counter (Perkin Elmer Corporation, Waltham, MA, USA). Urine, feces, and bile samples at each time period were mixed by equal percentages of either volume or weight. After the mixed samples were treated, the radio-profiles and metabolite identification in the bile, urine, and feces samples were carried out by LC-RAM and UPLC-Q Exactive HRMS, and the TRA% of each metabolite in plasma and the percentage of dose (dose%) of each compound in urine, feces, and bile samples were calculated.

#### 3.2.4. Identification of P450 Isozymes Responsible for Metabolism

Based on the result of metabolite identification, two metabolites were selected as targets for metabolic phenotyping analysis. Seven CYP isozymes (CYP1A2, CYP2B6, CYP2C8, CYP2C9, CYP2C19, CYP2D6, and CYP3A4) were investigated using the rhCYP isozymes method [22]. In brief, 7–16 (1 µΜ) was incubated with each rhCYP isozyme (20 pg/mL) in 100 mM sodium phosphate buffer (pH 7.4) containing 3 mM magnesium chloride. After 10 min of preincubation at 37 °C, the reactions were initiated by adding NADPH (1 mM) and then incubated at 37 °C in a shaking water bath for 0, 5, 15, and 30 min. The specific substrates of each rhCYP isozyme were incubated as the above method to confirm the activity of the incubation system. The samples were prepared in triplicates. The reactions were terminated by adding three times the volume of ice-cold acetonitrile (600 µL) and stored at −20 °C until analysis. After pre-treatment, the samples were determined by the LC–MS/MS (Shimadzu Exion-Triple Quad 4500) method. The relative contribution rate of each isozyme to the metabolism of 7–16 was evaluated in HLMs using the following formula: Contribution_x_% = V_x_ × Abundance_x_ (pmol/mg protein)/Σ(V_i_ × Abundance_i_). V_x_ (µL/min/pmol) is the production rate of metabolites for each rhCYP isozyme.

#### 3.2.5. Transporter Inhibitor Assay

The identification of potential inhibition of 7–16 on P-gp using the MDR1-MDCKII cell line, which is the Madin-Darby canine kidney cell line stably transfected with human MDR1 gene encoding the P-gp. The inhibition assay of 7–16 on other transports was conducted with recombinant stable HEK293 cell lines expressing human OATP1B1, OATP1B3, OAT1, OAT3, and OCT2, which were supplied by GenoMembrane (Kanagawa, Japan). The study was accomplished by WuXi AppTec Co., Ltd. (Shanghai, China) Briefly, cells were incubated in 5% CO_2_ at 37 ± 1 °C with saturated humidity, and the inhibition effect of 7–16 on the substrate uptake activity of each transporter are measured at 8 concentrations (0.045~100 μM).

For the P-gp inhibitor assay, MDR1-MDCKII cells at passage 8 were seeded on the 96-well insert system at the density of 2.3 × 10^5^ cells/cm^2^ and cultured for 6 days before being used in the transport experiment. After a 150 min transport experiment, the bi-directional permeability and efflux ratios of digoxin (10.0 µM), a known P-gp substrate, in the presence or absence of different concentrations of 7–16/verapamil (a positive inhibitor) were determined by an LC–MS/MS assay. The P-gp activity was represented by the efflux ratios of digoxin in the presence and absence of 7–16, and the %P-gp activity values of MDR1-MDCK II cells were used to calculate half maximal inhibitory concentration (IC_50_).

Similarly, HEK293-OATP1B1, OATP1B3, OAT1, OAT3, and OCT2 cells were seeded onto 96-well plates with a density of 1.5 × 10^5^ cells/cm^2^ and incubated for 24 h, then incubated with the relevant substrates in the absence and presence of 7–16 or corresponding positive inhibitors. After incubation, concentrations of each substrate in samples were analyzed by LC–MS/MS. The percentage of the mean transport activity values in the presence and absence of test compounds was used for the calculation of IC_50_.

#### 3.2.6. Data Analysis

Thermo Compound Discover software was used to find expected and unknown mark backgrounds, and a fragment ion search scoring model was also used to screen and identify metabolites of 7–16. GraphPad Prism 5 software was used to calculate inhibition IC_50_ values. SPSS Statistics 20.0 software was used for data processing and analysis. Data were expressed as mean ± standard deviation.

## 4. Conclusions

A total of 10 metabolites of 7–16 were identified in vitro and in vivo, and their relative abundances were accurately determined using UPLC–UV-HRMS and LC-RAM. The relative abundance of each metabolite in human liver microsomes could be covered by that observed in animal species. 7–16 was metabolized mainly through mono-oxidation (formed M470-2) and N-demethylation (formed M440) pathways in vivo. CYP3A4 isozyme was responsible for the primary metabolic reactions. The relative abundance of prototype drugs in rat plasma and urine accounted for most of the content. Meanwhile, M470-2 was the main metabolite detected in the plasma. The metabolite M440 was the predominant metabolite in feces and bile. Based on the data excreted through the bile and urine, the absorption rate of 7–16 was noted to be at least 74.7%. The potentiality to cause DDIs due to transporter inhibition by 7–16 was low. The comprehensive PK properties of 7–16 contributed to its further development of a new treatment for PDP, and these results should promote its clinical development.

## Figures and Tables

**Table 1:** Summary of the metabolites of 7–16 detected in human, mouse, rat, dog, and monkey liver microsomes using the UPLC-Q Exactive HRMS.

No.	RT	Theoretical	Experimental	Error	Formula	Relative Abundance, UV Area%				
	Min	Mass *m*/*z*	Mass *m*/*z*	ppm	[M + H]^+^	Human	Mouse	Rat	Dog	Monkey
M372	9.91	373.2034	373.2025	−2.4	C_20_H_26_FN_4_O_2_	0.60	0.61	1.10	1.73	2.52
M470-1	17.34	471.2014	471.2003	−2.3	C_22_H_27_F_4_N_4_O_3_	+	+	+	+	+
M438	19.85	439.1752	439.1743	−2.0	C_21_H_23_O_2_N_4_F_4_	0.20	1.06	0.99	1.53	0.76
M452	20.32	453.1908	453.1898	−2.2	C_22_H_25_F_4_N_4_O_2_	0.32	1.13	1.92	1.14	0.43
M440	21.02	441.1908	441.1903	−1.1	C_21_H_25_O_2_N_4_F_4_	0.78	0.39	1.29	0.47	1.73
7–16	21.15	455.2065	455.2054	−2.4	C_22_H_27_F_4_N_4_O_2_	95.2	91.3	86.1	77.2	90.6
M468	21.27	469.1857	469.1847	−2.1	C_22_H_25_F_4_N_4_O_3_	0.40	0.48	+	0.59	1.11
M470-2	22.02	471.2014	471.2007	−1.5	C_22_H_27_F_4_N_4_O_3_	2.53	5.05	8.63	17.3	2.81

RT: retention times; theoretical mass (*m*/*z*) = exact mass + 1.0073 (H^+^); exact mass: monoisotopic mass; error (ppm) = [(measured mass − theoretical mass) ÷ theoretical mass] × 10^6^; +: only detected by mass spectrometry.

**Table 2:** Summary of the metabolites of 7–16 detected in rat plasma, urine, feces, and bile obtained after a single oral dose of [^14^C]7–16 at 3 mg/100 µCi/kg plasma using UPLC-Q Exactive HRMS.

No.	RT	Theoretical	Experimental	Error	Formula	TRA% ^a^ (F/M), or Dose% ^b^			
	Min	Mass *m*/*z*	Mass *m*/*z*	ppm	[M + H]^+^	Plasma	Urine	Feces	Bile
M578	11.27	581.2428	581.2430	0.3	C_25_^14^CH_36_FN_6_O_6_S	−/−	−/−	−/−	1.60/2.32
M372	13.42	375.2066	375.2064	−0.5	C_19_^14^CH_26_O_2_N_4_F	1.11/2.50	4.34/5.75	4.97/5.42	0.41/0.83
M646	18.70	649.2367	649.2362	−0.8	C_27_^14^CH_35_O_9_N_4_F_4_	+/0.05	0.02/0.44	−/−	1.08/1.06
M456	27.92	459.1889	459.1887	−0.4	C_20_^14^CH_25_F_4_N_4_O_3_	+/0.20	2.12/4.94	0.72/0.42	+/0.05
M470-1	29.15	473.2046	473.2042	−0.8	C_21_^14^CH_27_O_3_N_4_F_4_	+/1.20	0.42/2.41	1.35/1.41	−/−
M438	37.86	441.1784	441.1784	−0.5	C_20_^14^CH_23_O_2_N_4_F_4_	2.51/1.50	0.02/0.02	0.32/0.58	0.10/0.25
M452	39.79	455.1940	455.1938	−0.4	C_21_^14^CH_25_O_2_N_4_F_4_	1.17/1.50	+/+	0.23/0.15	0.05/0.06
M440	41.79	443.1940	443.1935	−1.1	C_20_^14^CH_25_O_2_N_4_F_4_	1.40/3.70	2.66/5.77	21.1/24.2	2.71/3.83
[^14^C]7–16	42.82	457.2097	457.2092	−1.1	C_21_^14^CH_27_O_2_N_4_F_4_	81.9/57.3	23.6/9.79	16.8/5.29	0.76/0.53
M468	44.98	471.1889	471.1884	−1.1	C_20_^14^CH_25_O_3_N_4_F_4_	ND	0.02/0.58	−/−	0.24/0.20
M470-2	46.42	473.2046	473.2046	−0.0	C_21_^14^CH_27_O_3_N_4_F_4_	8.63/27.2	2.50/1.21	−/−	2.39/2.86

RT: retention times; theoretical mass (*m*/*z*) = exact mass + 1.0073 (H^+^); exact mass: monoisotopic mass; error (ppm) = [(measured mass − theoretical mass) ÷ theoretical mass] × 10^6^; TRA: total radiation intensity; F: female; M: male; −: not detected; +: only detected by mass spectrometry; ^a^, TRA% in plasma; ^b^, Dose% in urine, feces, and bile.

**Table 3:** Inhibitory effect of 7–16 on P-gp, OATP1B1, OATP1B3, OAT1, OAT3, and OCT2 transporters.

Transporters	Substrates	Inhibition IC_50_ (μM)	
		7–16	Control (Positive Inhibitor)
P-gp	Digoxin	30.6	1.57 (Verapamil)
OATP1B1	Estrone 3-sulfate	>100	0.0556 (Cyclosporin A)
OATP1B3	β-Estradiol 17-(β-d-glucuronide)	89.5	0.734 (Ritonavir)
OAT1	p-Aminohippurate	>100	3.80 (Probenecid)
OAT3	Estrone 3-sulfate	>100	1.43 (Probenecid)
OCT2	Metformin	72.5	11.3 (Verapamil)

P-gp: P-glycoprotein; OATP1B1: organic anion transporting polypeptide 1b1; OATP1B3: organic anion transporting polypeptide 1b3; OAT1: organic anion transporter 1; OAT3: organic anion transporter 3; OCT2: organic cation transporter 2.

## Data Availability

Data are contained within the article.
